# Assessment and Improvement of Quality of Life in Patients Undergoing Treatment for Head and Neck Cancer

**DOI:** 10.7759/cureus.1215

**Published:** 2017-05-02

**Authors:** Aamina Majid, Bushra Z Sayeed, Maryam Khan, Murk Lakhani, Mariam M Saleem, Hina Rajani, Priyanka Ramesh, Nauman Hashmani, Muneeba Zia, Husnain Abid, Bushra Majid, Momal Jamali, Kinza Murtaza, Aneeqa Kamal, Maryam Hussain

**Affiliations:** 1 Student, Dow Medical College, Karachi, Pakistan; 2 Dow Medical College, Dow University of Health Sciences (DUHS), Karachi, Pakistan

**Keywords:** oncology, head and neck cancer, quality of life

## Abstract

Introduction: We compared the pre and post-treatment quality of life in head and neck cancer (HNC) patients and identified factors that could improve the quality of life in such patients.

Methods: The European Organization for Research and Treatment of Cancer Quality of Life Questionnaire C30 (EORTC QLQ-C30) questionnaire was administered to 84 patients’ pre and post-treatment. Patients who had non-metastatic, measurable, and untreated HNCs were invited, provided that their age was below 80 years. We did not discriminate based on treatment modality, stage of cancer, or co-morbidities. Patients who were mentally incapacitated, with secondary or recurrent HNC, distant metastasis, skin cancer, congenital anomaly of the head and neck, chronic illness, or any previous or current psychiatric illness were excluded from the study. A high mean score on the functional scale and a low score on symptom scale signify a better quality of life. We used the dependent t-test to compare pre and post-treatment scores.

Results: We found no statistically significant differences in any variables, except the four symptom scales of diarrhoea, constipation, nausea/vomiting, and financial difficulty. All of these variables had increased mean scores with p values of < 0.001. Also, we found no statistical significance (p = 0.250) when comparing the pre-treatment (59.4 ± 18.3) and post-treatment (61.2 ± 16.2) scores for the global health status.

Conclusion: We found no improvement in the quality of life in HNC patients despite intervention. In fact, diarrhoea, constipation, nausea/vomiting, and financial difficulty of these patients worsened post-treatment.

## Introduction

Cancer is a leading cause of death worldwide after heart diseases [1]. Head and neck cancer (HNC), an umbrella term for malignancies of larynx and hypopharynx, nasal cavity, paranasal sinuses, nasopharynx, oropharynx, oral cavity, and salivary gland, accounts for about half a million cases annually, ranking it as the sixth most common cancer globally [2]. About 90% of HNCs are squamous cell carcinomas (HNSCC) arising from the epithelium in the region of head and neck after exposure to carcinogens, such as tobacco, smoking, and alcohol [3]. In developing countries like Pakistan, carcinoma of the oral mucosa is the most commonly diagnosed HNSCC with males having a greater preponderance, attributed to the local custom of excessive use of cigarettes, tobacco, betel nut, and areca nut. Due to advancements in diagnostic and treatment modalities, the survivorship of HNC patients has increased significantly during the last decade [1]. However, these remarkable but aggressive treatment methods also bring along numerous side effects that significantly affect the quality of life (QoL) of the patients.

QoL, also known as the patient’s perception of his/her general well-being, is a multidimensional concept that includes psychological, social, occupational, functional, and physical well-being [4]. The term health-related QoL (HRQoL) is preferred over QoL as it only focuses on the health status and disease-related issues, such as symptoms and functions [5]. Due to the anatomic complexity and functional importance of the head and neck region, patients of HNC face multiple challenges pre and post-treatment, such as dysphagia, pain, xerostomia, dietary restrictions, and physical restrictions besides disfigurement and problems in sexual life [4, 6]. Therefore, apart from survivorship, the assessment of HRQol has become imperative for optimum patient-centred decision-making.

Assessment of the HRQoL of HNC patients and its relationship with various demographic and disease variables remains a neglected area of research in Pakistan [7-8], and thus, less emphasis has been laid upon improving the QoL and returning the patient to his pre-illness functional state. Therefore, the primary objectives of this study were to measure the HRQoL and to evaluate factors that could improve the QoL of HNC patients undergoing treatment. Besides this, the secondary objective was to exclusively highlight the impact on separate domains of HRQoL, an important survival outcome that can guide healthcare professionals to tailor treatment and rehabilitation according to expected functional outcomes.

## Materials and methods

A prospective cohort study was conducted at the Civil Hospital Karachi (CHK) after approval from the Institutional Review Board of Dow University Health Sciences. The study was carried out during the period of January 1, 2016 to June 30, 2016.

Although HNC is more common among males [9], we decided to select both males and females for our study. A total of 84 patients with non-metastatic, measurable, and untreated cancer of the oral cavity were invited consecutively to participate in this QoL study, with patients’ age being less than 80 years of age. The patients were selected irrespective of whether they were receiving either radiotherapy, chemotherapy, or a combination of both as the treatment modality with curative intent. We recruited the patients regardless of the stage of cancer they were suffering from and the comorbidities that they already had. Patients who were mentally incapacitated, with secondary or recurrent HNC, distant metastasis, skin cancer, congenital anomaly of the head and neck, chronic illness, or any previous or current psychiatric illness were excluded from the study. Those who were unable to answer the questionnaire due to senile dementia or severe intercurrent disease were also excluded.

Both written and verbal consent were taken from the patients before interviewing them. The patients were requested to come to CHK where they were asked to fill out the questionnaire by trained interviewers before (baseline) and between three to six months after undergoing the treatment. Two trained interviewers conducted the interviews in order to eliminate any chances of interviewer bias. The interviewers were well versed with the major languages of Pakistan, namely English, Urdu, Sindhi, and Pushto, to remove any language barrier between the interviewer and the patient and to ensure that all eligible patients were included.

The QoL of patients was evaluated by means of the European Organization for Research and Treatment of Cancer Quality of Life Questionnaire C30 (EORTC QLQ-C30) [10]. The multicultural validity and psychometric scales established on the basis of this questionnaire are considered satisfactory [10-13]. With the assistance of the EORTC QLQ-C30, 17 scales were extracted from the first 30 items. These scales included five functional scales (strenuous physical activity, mild physical activity, emotional involvement, cognitive functioning, and social interaction), three symptom scales (lethargy, nausea and vomiting, and pain), six single-item symptom scales (dyspnoea, insomnia, decreased appetite, constipation, diarrhoea, and financial problems), a scale for depression and amnesia, and one global health status/QoL scale. Greater scores in functioning and global health status/QoL are indicative of a higher level of functioning and a better Q0L, respectively, whereas higher scores in the symptom scale amount to a higher level of symptomatology and a poor QoL.

Data was entered and analysed using the Statistical Package for the Social Sciences (SPSS) v23.0 (IBM SPSS Statistics, Armonk, NY). For categorical variables, frequencies and percentages were reported while for continuous variables, mean and standard deviation were reported. Comparison of continuous variables was done using dependent T-test. 

## Results

### General characteristics

A total of 84 patients were included in the study with the majority being male (n = 76; 90.5%). More than two-thirds were married (n = 65; 77.4%), had a low income (n = 73; 86.9%), and had received primary/secondary education (n = 61; 72.6%). The most prevalent primary tumour site was the oral cavity (n = 54; 64.3%) and nearly three-fifths of the total patients received chemotherapy and radiotherapy combined (n = 51; 60.7%). Around one-third of the patients were diagnosed as Stage IV (n = 32; 38.1%). A great majority were smokers, followed by betel nut chewers and alcohol consumers (n = 79, 94.0%; n = 72, 85.7%; n = 53, 63.1%, respectively). More than one-third of the patients suffered from diabetes as well (n = 37; 44.0%). The general and demographic characteristics of the patients are summarized in Table 1. 

**Table 1:** The Baseline Characteristics of the Patients

Patient Characteristics	No of Patients	Percent (%)
	(n = 84)	
Sex		
Male	76	90.5
Female	8	9.5
Age, years		
≤ 60	49	58.3
> 60	35	41.7
Marital Status		
Married	65	77.4
Unmarried	19	22.6
Income		
Low-income	73	86.9
Level of education		
Primary/Secondary	61	72.6
Treatment Modality		
Chemotherapy + Radiation	51	60.7
Stage of cancer		
Stage IV	32	38.1
Site of cancer		
Oral cavity	54	64.3
Smokers	79	94
Betel nut chewers	72	85.7
Alcohol consumers	53	63.1
Co-morbidities		
Diabetics	37	44

### EORTC QLQ C30 score comparisons

We found no statistical significance (p = 0.250) when comparing the pre-treatment (59.4 ± 18.3) and post-treatment (61.2 ± 16.2) scores for the global health status. Similarly, no significance was found in any of the pre-treatment and post-treatment functional scales: Physical (83.2 ± 14.5 to 84.1 ± 13.9, p = 0.341), emotional (67.4 ± 19.7 to 68.1 ± 20.3, p = 0.410), social (81.3 ± 19.9 to 77.3 ± 19.2, p = 0.907), role (73.8 ± 20.2 to 72.6 ± 20.7, p = 0.648), and cognitive (90.5 ± 16.3 to 90.2 ± 15.9, p = 0.548).

After completion of treatment, a statistically significant increment in the complaints of diarrhoea (1.6 to 6.8), constipation (1.4 to 6.4), and nausea/vomiting (8 to 12.3) was observed (p values = < 0.001). There was also a slight increase in the mean scores of fatigue, dyspnoea, and loss of appetite after treatment. An improvement was only seen in insomnia (p = 0.995) and pain (p = 0.959); however, none of these reached statistical significance. Financial difficulty increased after treatment (43.6 to 58.2) and was statistically significant (p = < 0.001). Table 2 presents the mean scores and standard deviations of various scales and single items.

**Table 2:** Scores of EORTC QLQ C30 Among HNC Patients Pre and Post-treatment EORTC QLQ C30: European Organization for Research and Treatment of Cancer Quality of Life Questionnaire C30; HNC: head and neck cancer; SD: Standard deviation

Scales and Items	Pre-treatment		Post-treatment		P
	Mean	SD*	Mean	SD*	
Global Quality of Life	59.4	18.3	61.2	16.2	0.25
Functional Scales					
Physical	83.2	14.5	84.1	13.9	0.341
Emotional	67.4	19.7	68.1	20.3	0.41
Social	81.3	19.9	77.3	19.2	0.907
Role	73.8	20.2	72.6	20.7	0.648
Cognitive	90.5	16.3	90.2	15.9	0.548
Symptom Scales					
Fatigue	27.3	15.4	29.7	13.6	0.143
Pain	29	19.2	24.2	16.3	0.959
Dyspnoea	9.1	17.1	9.7	18	0.412
Insomnia	37.6	20.3	30	17.2	0.995
Loss of appetite	26.2	13.4	29.1	14.2	0.087
Diarrhoea	1.6	6.2	6.8	9.8	<0.001
Constipation	1.4	4.2	6.4	7.3	<0.001
Nausea/vomiting	8	6.4	12.3	7.1	<0.001
Financial difficulty	43.6	20.1	58.2	23.2	<0.001

## Discussion

Our results suggest that there was no statistically significant change in the HRQoL of our patients as assessed prior to the treatment and post-treatment. A literature review shows variable results regarding the HRQoL of HNC patients. Some studies reported that HRQoL returns to the pre-treatment score while others showed a decline or increase in the score [14-16]. Most of our patients had concurrent chemoradiation therapy (CCRT) as it has a better survival rate for locally advanced stages as compared to radiotherapy alone [17-18]. Therefore, the modality of the treatment cannot be the reason for the insignificant change in HRQoL in this study.

We found a significant male preponderance (90%), with the majority being under 60 years of age and of low socioeconomic status. Most of them were smokers, betel nut chewers, or alcoholics, which may have a strong correlation with the predominant existence of oral cavity cancer in our study. These demographic characteristics of the observed populace are in correspondence with the known risk factors of HNC [19-21].

Our study also shows an increased incidence in those belonging to low socioeconomic status, which might be a major reason for emotional instability and, hence, resulting in increased psychological stress. This suggests that psychological stress is a key factor leading to the consumption of tobacco, betel nut, or alcohol [22]. Moreover, our data shows that the majority of our patients are literate; however, literacy does not necessarily equate to awareness [23]. Therefore, there is a dire need to spread this knowledge about the preventable/avoidable risk factors of the HNC.

No statistically significant improvement was seen in the global quality of life, functional scale, and symptom scale, but there is observable variation in the individual components. There was some improvement in the physical and emotional function while the other three variables decreased post-treatment, including role performance, cognitive, and social function. It is likely due to post-treatment supportive care factors. A feeding tube after the treatment can be associated with low role performance and social function [24], for example. Contrary to our results, however, the literature review expressed an increase in mental health after treatment because of depression and anxiety due to the cancer diagnosis before the treatment [24-25].

A major factor for the negative outcome of this study was the presentation of the patient at an advanced stage of the disease. In order to address this issue, patients and healthcare providers need to work hand in hand. A vast majority of patients neglect oral lesions due to its silent presentation, lack of education, lack of resources, or misdiagnosis. Therefore, it is vital for healthcare providers to focus on screening the high-risk population for pre-malignant features and arrange health awareness programs regarding risk factors, prevalence, and early presenting features of HNC. Also, it is important to counsel cancer patients regarding treatment modalities, support facilities, and follow-up plans for improvement in physical as well as psychological QoL.

Most of the HNC patients in our study were habitual betel nut chewers (85.7%). Studies suggest that HNC patients with betel nut chewing as a risk factor express different mutations than those who were not addicted to betel nuts [26-27]. Consequently, these patients respond to treatment modality in a separate manner [27-28]. Therefore, further evaluation should be considered to assess and compare the effect of individual treatment modalities in relation to causative agents of the cancer.

There are a few limitations in this study which need to be considered. Firstly, this study was administered using a validated questionnaire EORTC QLQ C30. However, it would have yielded more specific information if we would have employed an additional cancer site-specific EORTC QoL module of head and neck cancer (QLQ-H&N43 questionnaire). Secondly a longer follow-up time period would have broadened our horizon of understanding even more.

## Conclusions

This study emphasizes that HNC patients’ HRQoL can express similar scores before and after the treatment. As for significant changes post-treatment, we observed worsening in the frequency of diarrhoea, nausea/vomiting, constipation, and financial difficulty. For future studies, efficacy can be improved by considering the long-term follow-up assessment and treatment modality according to causative agents affecting the HRQoL in these patients.
